# Fine-Tuning Circadian Rhythms: The Importance of *Bmal1* Expression in the Ventral Forebrain

**DOI:** 10.3389/fnins.2017.00055

**Published:** 2017-02-09

**Authors:** Michihiro Mieda, Emi Hasegawa, Nicoletta Kessaris, Takeshi Sakurai

**Affiliations:** ^1^Department of Molecular Neuroscience and Integrative Physiology, Graduate School of Medical Sciences, Kanazawa UniversityKanazawa, Japan; ^2^Department of Cell and Developmental Biology, Wolfson Institute for Biomedical Research, University College LondonLondon, UK

**Keywords:** bmal1, hypothalamus, *Nkx2.1*, circadian rhythm, sleep-wakefulness regulation, food intake

## Abstract

Although, the suprachiasmatic nucleus (SCN) of the hypothalamus acts as the central clock in mammals, the circadian expression of clock genes has been demonstrated not only in the SCN, but also in peripheral tissues and brain regions outside the SCN. However, the physiological roles of extra-SCN circadian clocks in the brain remain largely elusive. In response, we generated *Nkx2.1-Bmal1*^−/−^ mice in which *Bmal1*, an essential clock component, was genetically deleted specifically in the ventral forebrain, including the preoptic area, nucleus of the diagonal band, and most of the hypothalamus except the SCN. In these mice, as expected, PER2::LUC oscillation was drastically attenuated in the explants of mediobasal hypothalamus, whereas it was maintained in those of the SCN. Although, *Nkx2.1-Bmal1*^−/−^ mice were rhythmic and nocturnal, they showed altered patterns of locomotor activity during the night in a 12:12-h light:dark cycle and during subjective night in constant darkness. Control mice were more active during the first half than the second half of the dark phase or subjective night, whereas *Nkx2.1-Bmal1*^−/−^ mice showed the opposite pattern of locomotor activity. Temporal patterns of sleep-wakefulness and feeding also changed accordingly. Such results suggest that along with mechanisms in the SCN, local *Bmal1*–dependent clocks in the ventral forebrain are critical for generating precise temporal patterns of circadian behaviors.

## Introduction

The circadian oscillator in the suprachiasmatic nucleus (SCN) of the hypothalamus is the central pacemaker in mammals, orchestrating multiple circadian rhythms in organisms (Reppert and Weaver, 2002; Dibner et al., 2010). Each SCN cell has an individual cellular clock driven by autoregulatory transcriptional and translational feedback loops (TTFLs) of clock genes. Notably, such cellular clocks also appear in many peripheral tissues and brain regions outside the SCN (Balsalobre et al., 1998; Yamazaki et al., 2000; Yagita et al., 2001; Abe et al., 2002; Yoo et al., 2004; Guilding et al., 2009; Dibner et al., 2010). Cell type-specific manipulations of clock genes have clarified the roles of peripheral circadian clocks in a variety of tissues (McDearmon et al., 2006; Storch et al., 2007; Lamia et al., 2008; Westgate et al., 2008; Dibner et al., 2010; Marcheva et al., 2010; Sadacca et al., 2011; Fustin et al., 2012; Paschos et al., 2012; Nguyen et al., 2013; Gibbs et al., 2014; Jacobi et al., 2015; Xie et al., 2015; Dudek et al., 2016; Xu et al., 2016). By contrast, knowledge of the physiological roles of brain clocks in extra-SCN regions remains limited (Roybal et al., 2007; Mukherjee et al., 2010; Mieda and Sakurai, 2011; Spencer et al., 2013; Yu et al., 2014; Nakano et al., 2016; Orozco-Solis et al., 2016; Shimizu et al., 2016).

The hypothalamus plays a pivotal role in homeostasis and in controlling multiple bodily functions that exhibit circadian fluctuations, including sleep and wakefulness, body temperature, food intake, and autonomic nervous and endocrine systems. In addition, local brain clocks have been reported in multiple nuclei of the hypothalamus (Abe et al., 2002; Mieda et al., 2006; Guilding et al., 2009; Moriya et al., 2009; Yu et al., 2014; Orozco-Solis et al., 2016). Therefore, it is especially intriguing to pinpoint the roles of extra-SCN brain clocks in the hypothalamus.

To genetically dissect the roles of SCN and extra-SCN clocks, we used mice in which the expression of Cre recombinase was restricted to the hypothalamus outside the SCN. We focused on the developmental regulatory genes involved in the patterning of the neuroepithelium, which are often expressed in domains that give rise to certain brain regions and nuclei in adults. *Nkx2.1* is a good candidate because its expression affects a domain that generates the posteroventral hypothalamus, yet is devoid of the *Lhx1*-positive domain that differentiates into the SCN and its adjacent structures (Puelles and Rubenstein, 2003; Shimogori et al., 2010). Along with the neuroepithelial domain that gives rise to the posteroventral hypothalamus, *Nkx2.1* is also expressed developmentally in the medial ganglionic eminence (MGE) and anterior entopeduncular area (AEP) in the ventral forebrain, from which γ-aminobutyric acid (GABA) neurons originate and migrate into the cerebral cortex and basal ganglia, including the striatum and globus pallidus (Puelles and Rubenstein, 2003; Kessaris et al., 2006). The MGE and AEP also generate oligodendrocyte progenitors in developing cortical and subcortical structures, although those cells decline to a very small fraction of all oligodendrocytes in most parts of the adult forebrain (Kessaris et al., 2006).

In our study, we generated mice in which *Bmal1*, an essential transcription factor of the TTFL, was deleted specifically in cells marked by the developmental expression of *Nkx2.1*. We demonstrated that although the mice maintained nocturnality and a normal free-running period of behavioral rhythm driven by the intact SCN, the temporal patterns of their nocturnal locomotor activity, sleep-wakefulness cycle, and food intake were impaired.

## Methods

### Animals

To generate *Nkx2.1-Bmal1*^−/−^ mice (*Nkx2.1-Cre;Bmal1*^*fl*/−^), *Nkx2.1-Cre* mice (*N* > 7 backcrossed to C57BL/6J; Kessaris et al., 2006) were mated with mice carrying a conditional *Bmal1* allele (*Bmal1*^*fl*^; Storch et al., 2007) (*N* > 7 backcrossed to C57BL/6J, The Jackson Laboratory #007668), as well as with mice carrying a null *Bmal1* allele (*Bmal1*^−^) generated from *Bmal1*^*fl*^ mice (Mieda and Sakurai, 2011). *Nkx2.1-Bmal1*^−/−^ and control mice were further mated with *Per2::Luc* reporter mice (Yoo et al., 2004) in order to obtain *Nkx2.1-Bmal1*^−/−^;*Per2::Luc* (*Nkx2.1-Cre;Bmal1*^*fl*/−^;*Per2::Luc*) and Control;*Per2::Luc* (*Bmal1*^*fl*/−^;*Per2::Luc*) mice. Mice were maintained under a strict 12-h light:dark (LD) cycle in a temperature- and humidity-controlled room and fed *ad libitum*. All experimental procedures involving animals were approved by the respective animal care and use committee of Kanazawa University and were in accordance with the guidelines of the National Institutes of Health.

### Histological study

To examine the specificity of Cre-mediated recombination, *Nkx2.1-Cre* mice were crossed with *Rosa26R-lacZ* reporter mice (Jackson Laboratory #003474) (Soriano, 1999). Animals were sacrificed by transcardial perfusion with PBS followed by 4% paraformaldehyde fixative. Serial coronal brain sections 30 μm thick were collected in four series, one of which was further stained by β-galactosidase enzymatic labeling as previously described (Kessaris et al., 2006). Immunostaining was performed as previously reported (Mieda et al., 2015) with rabbit anti-BMAL1 (Novus Biologicals, 1:10,000), biotinylated anti-rabbit antibody (Vector Labs, 1:500), and the Vectastain ABC kit (Vector Labs).

### Bioluminescence measurement

Male *Nkx2.1-Bmal1*^−/−^;*Per2::Luc* (*n* = 6) and control;*Per2::Luc* mice (*Bmal1*^*fl*/−^;*Per2::Luc*; *n* = 5), aged 17–19 weeks, were housed in LD before sampling. PER2::LUC bioluminescence in SCN tissue was measured with a photomultiplier tube (Atto, Kronos Dio) at 10-min intervals with an exposure time of 1 min. Coronal SCN slices of 300 μm were made with a vibratome (Leica, Vi1000S) at approximately ZT8–10. The SCN tissue was dissected at the midrostrocaudal region, and a paired SCN was cultured on a Millicell-CM culture insert (Millipore), as previously described (Mieda et al., 2015). Bilateral MBH explants containing the dorsomedial (DMH), ventromedial (VMH), and arcuate (Arc) nuclei of the hypothalamus, as well as the median eminence (ME) and pars tuberalis (PT) (Guilding et al., 2009) were cultured similarly to the SCN explant. Recorded values were detrended by subtracting 24-h moving average values and were smoothened with a 5-point moving average method. Because luminescence from the SCN of *Nkx2.1-Bmal1*^−/−^;*Per2::Luc* mice was weak and its waveform was noisy, the middle of the time points crossing value 0 upward and downward were defined as acrophases, and the intervals between two adjacent acrophases were calculated for the periods. The average of periods of initial three cycles was calculated for each explant.

### Measurements of locomotor activity and food intake

Male *Nkx2.1-Bmal1*^−/−^ (*Nkx2.1-Cre*;*Bmal1*^*fl*/−^; n = 11) and control (*Bmal1*^*fl*/−^ and *Nkx2.1-Cre;Bmal1*^+/*fl*^; *n* = 8 and 6, respectively) mice, aged 9–15 weeks, were housed individually in cages placed in a light-tight chamber with a light intensity of ~50 lux. Spontaneous locomotor activity and food intake were recorded by infrared motion sensors and food intake monitor (O'Hara) in 10-min bins. Actogram, activity profile, and χ^2^ periodogram analyses were performed using ClockLab (Actimetrics). The free-running period was measured by periodogram for Days 5–21 in constant darkness (DD).

### Sleep recordings

This study used male *Nkx2.1-Bmal1*^−/−^ (*Nkx2.1-Cre*;*Bmal1*^*fl*/−^; *n* = 4) and control (*Bmal1*^*fl*/−^ and *Nkx2.1-Cre;Bmal1*^+/*fl*^; *n* = 3 for each genotype; i.e., 6 control mice in total) mice, aged 17–21 weeks. These mice were different from those used for measurements of locomotor activity and food intake. The implantation of an electroencephalogrammic (EEG) and electromyographic (EMG) electrode was performed as described previously (Sasaki et al., 2011). Following surgery, all animals were housed individually for a recovery period of at least 14 days, after which EEG-EMG recordings were performed on 3 consecutive days in LD. EEG-EMG data were analyzed as previously described (Sasaki et al., 2011).

### Statistics

All results are expressed as the mean ± SEM. Comparisons between individuals were analyzed by a two-tailed Student's *t*-test. In Figure S1, an one-way repeated measure ANOVA was performed, followed by a Tukey-HSD *post-hoc* analysis. When appropriate, data were analyzed by a two-way repeated measure ANOVA, followed by a Tukey-HSD *post-hoc* test.

## Results

### Cre-mediated recombination in the hypothalamus of *Nkx2.1-Cre* mice

**Figure 1:**
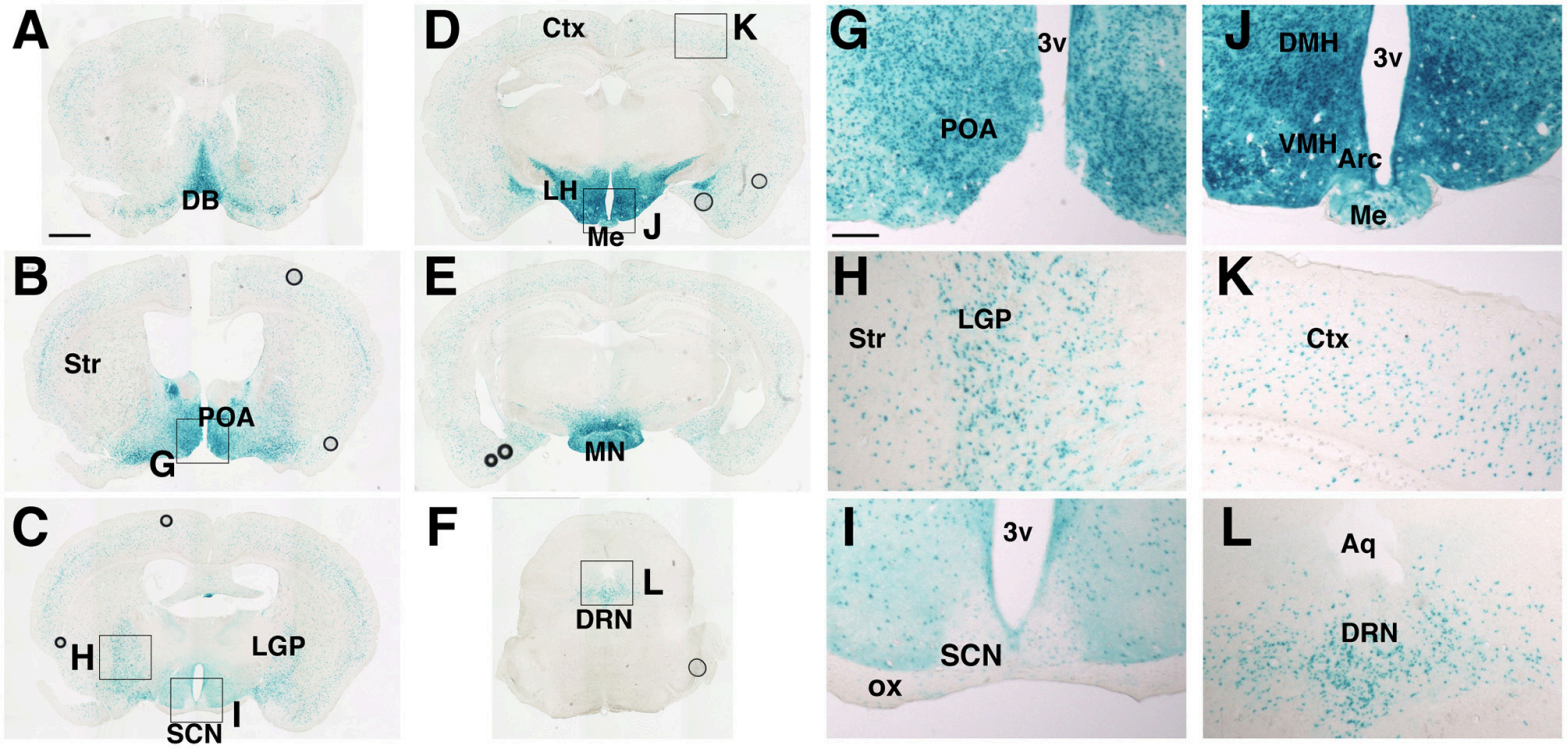
**Distribution of cells with Cre-mediated recombination in ***Nkx2.1-Cre*** mice crossed with ***Rosa26R-lacZ*** reporter mice**. Coronal sections from the olfactory bulb to medulla were stained by β-galactosidase enzymatic labeling. **(A–F)** Representative images with positive cells. **(G–L)** Magnified images of regions in **(B–F)**. Scale bars, 1 mm for **(A–F)**; 200 μm for **(G–L)**. ac, anterior commissure; 3v, Arc, arcuate hypothalamic nucleus; Aq, aqueduct; Ctx, cerebral cortex; DB, diagonal band; DMH, dorsomedial hypothalamic nucleus; DRN, dorsal raphe nucleus; LGP, lateral globus pallidus; LH, lateral hypothalamus; Me, medial eminence; MN, mammillary nuclei; POA, preoptic area; SCN, suprachiasmatic nucleus; Str, striatum; VMH, ventromedial hypothalamic nucleus (Paxinos and Franklin, 2001).

According to the prosomeric model (Puelles and Rubenstein, 2003), the SCN and its adjacent areas develop from a field dorsal to the anterior basal floor of the secondary prosencephalon, which generates most areas of the hypothalamus and is delineated by the expression of the homeodomain transcription factor Nkx2.1. Therefore, we examined *Nkx2.1-Cre* mice (Kessaris et al., 2006) as a candidate Cre driver, in which Cre is expressed specifically in the hypothalamus but not the SCN. To localize cells with Cre-mediated recombination, *Nkx2.1-Cre* mice were crossed with *Rosa26R-lacZ* reporter mice, which expressed β-Galctosidase (βGal) after the Cre-mediated deletion of a *loxP*-flanked transcriptional blocker (Soriano, 1999). We mapped βGal+ cells in the entire brain of adult mice (Figure 1). Prominent βGal expression was observed in the medial septum, nucleus of the diagonal band (Figure 1A), medial preoptic area (Figures 1B,G), and most areas of the hypothalamus: the ventromedial (VMH), dorsomedial (DMH), arcuate (Arc) (Figures 1D,J), mammillary nuclei (MN) (Figure 1E), and the lateral hypothalamic area (LHA) at the rostrocaudal level of and posterior to the VMH (Figure 1D). Notably, the SCN, PVH, and adjacent regions were nearly devoid of βGal+ cells (Figures 1C,I). Moreover, βGal expression was detected in cells scattered in the cerebral cortex (Figure 1K), striatum, and globus pallidus (Figure 1H), which were likely GABAergic neurons originated from the Nkx2.1+ MGE and AEP. Such expression was entirely consistent with the developmental expression of Cre recombinase in *Nkx2.1-Cre* mice (Kessaris et al., 2006). We also detected several βGal+ cells in the dorsal raphe nucleus (Figures 1F,L). Altogether, *Nkx2.1-Cre* mice may be useful in revealing the roles of extra-SCN clocks in the hypothalamus.

### PER2::LUC oscillation attenuated in the mediobasal hypothalamus of *Nkx2.1-Bmal1*^−/−^ mice

**Figure 2:**
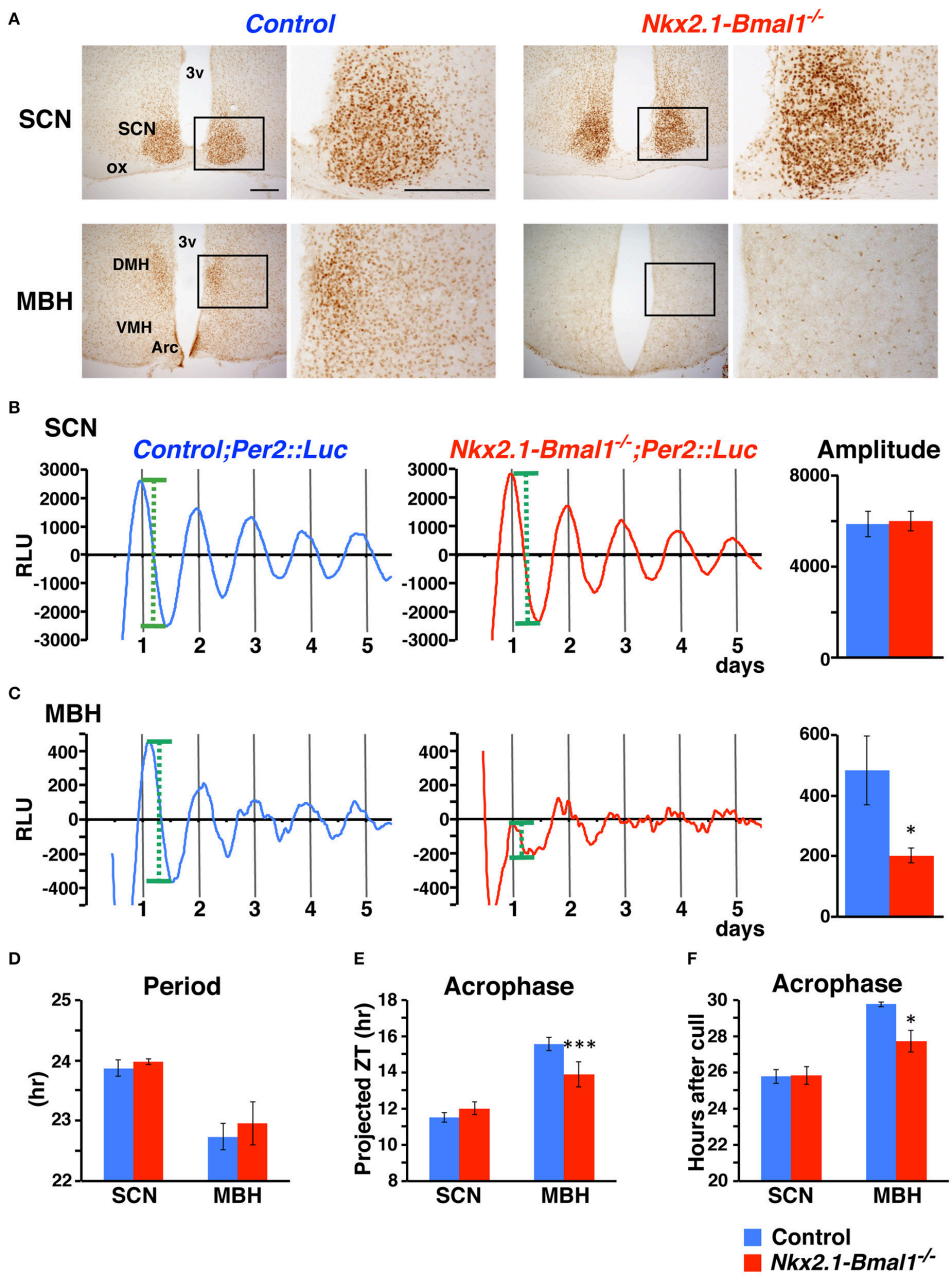
**PER2::LUC oscillation is attenuated in the mediobasal hypothalamus but not in the SCN of ***Nkx2.1-Bmal1***^**−/−**^ mice. (A)** BMAL1 expression in the SCN and mediobasal hypothalamus (MBH) of control and *Nkx2.1-Bmal1*^−/−^ mice. Coronal brain sections were immunostained for BMAL1. The locations of the magnified images are indicated by rectangles in the low-power images. Scale bars, 200 μm. **(B,C)** Representative circadian rhythm of PER2::LUC in slices of the SCN **(B)** and MBH **(C)** of control and *Nkx2.1-Bmal1*^−/−^ mice housed in LD. Mean amplitudes of PER2::LUC oscillation are shown on the right. Definition of amplitude was demonstrated by green dotted lines in **(B,C)**. **(D–F)** Mean periods **(D)** and first acrophases **(E,F)** of PER2::LUC oscillation. Because PER2::LUC expression in the MBH was reported to be significantly correlated with time of cull (Guilding et al., 2009), the first acrophases were also expressed in hours after cull **(F)**. Two-way repeated measures ANOVAs revealed statistical significances in the effect of brain region for period [*F*_(1, 8)_ = 17.16, *p* = 0.003], in the effects of genotype [*F*_(1, 8)_ = 619.19, *p* < 0.001] and region [*F*_(1, 8)_ = 230.96, *p* < 0.001], as well as in the interaction between genotype and region [*F*_(1, 8)_ = 339.05, *p* < 0.001] for first acrophase (projected ZT), and in the effects of genotype [*F*_(1, 8)_ = 6.92, *p* = 0.030] and region [*F*_(1, 8)_ = 22.27, *p* = 0.002] for first acrophase (hours after cull). Values are mean ± SEM. ^*^p < 0.05; ^***^p < 0.001; comparison between genotypes by a Tukey-HSD *post-hoc*. *n* = 5 for control;*Per2::Luc, n* = 6 for *Nkx2.1-Bmal1*^−/−^;*Per2::Luc* mice.

We generated mice without the *Bmal1* gene in the hypothalamus, except for in the SCN, by breeding *Nkx2.1-Cre* mice with mice carrying floxed *Bmal1* alleles (*Bmal1*^*fl*^; Storch et al., 2007). To improve the efficiency of *Bmal1*^*fl*^ allele deletion, we generated *Nkx2.1-Cre;Bmal1*^*fl*/−^ mice to have one floxed and one null allele of the *Bmal1* gene (Mieda and Sakurai, 2011), hereafter designated as *Nkx2.1-Bmal1*^−/−^ mice. We first confirmed in these mice that BMAL1 expression was drastically reduced in the ventral forebrain, including the Arc, VMH, and the DMH, but not in the SCN and adjacent regions (Figure 2A). Before analyzing their behavior in detail, we further crossed *Nkx2.1-Cre* mice with mice carrying the PER2::LUC reporter (Yoo et al., 2004) to confirm *ex vivo* that circadian clocks in the mediobasal hypothalamus (MBH; Guilding et al., 2009) were functionally attenuated in *Nkx2.1-Bmal1*^−/−^ mice. Coronal slices were prepared from adult mice, and PER2::LUC oscillation in the SCN of *Nkx2.1-Bmal1*^−/−^ mice was similar to that of control mice (amplitude: 6002 ± 435 vs. 5872 ± 559, *p* = 0.857; Figure 2B). By contrast, PER2::LUC oscillation in the MBH of *Nkx2.1-Bmal1*^−/−^ mice was far less robust and unstable with reduced amplitude (amplitude: 202 ± 24 vs. 482 ± 113, *p* = 0.018; Figure 2C). The values of period and first acrophase of PER2::LUC oscillation in control explants were similar to those reported previously in both the SCN and MBH (Figures 2D–F; Yoo et al., 2004; Guilding et al., 2009). Interestingly, the first acrophase of PER2::LUC oscillation in the MBH occurred earlier in *Nkx2.1-Bmal1*^−/−^ mice than control mice. Thus, local circadian clocks in the MBH were severely impaired in *Nkx2.1-Bmal1*^−/−^ mice, whereas the SCN central clock remained normal.

### Altered temporal pattern of nocturnal locomotor activity in *Nkx2.1-Bmal1*^−/−^ mice

**Figure 3:**
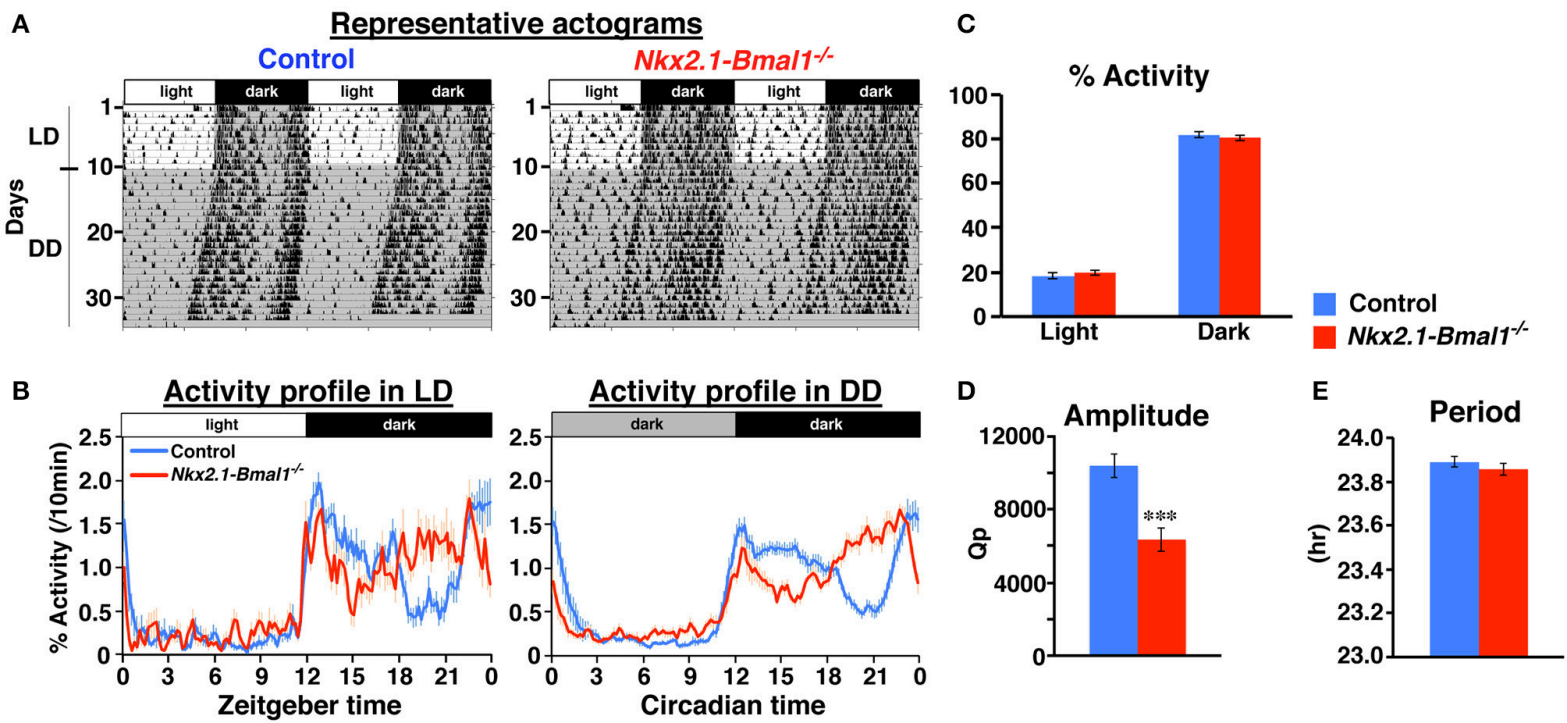
**Temporal pattern of nocturnal locomotor activity is altered in ***Nkx2.1-Bmal1***^**−/−**^**. **(A)** Representative locomotor activity of control and *Nkx2.1-Bmal1*^−/−^ mice. Animals were initially housed in 12:12-h light/dark (LD) conditions and later transferred to constant darkness (DD). Gray shading indicates when lights were off. **(B)** Daily profile of locomotor activity in LD or DD. Activity counts are expressed as percentage of daily total. **(C)** Relative activity during 12-h light phase (Light) and 12-h dark phase (Dark). No significant difference was detected between genotypes. **(D,E)** Mean free-running period and amplitude (Qp values of periodogram) in DD. Values for activity in DD were calculated for data on Days 5–19 in DD. Values are mean ± SEM. *n* = 14 for control, *n* = 11 for *Nkx2.1-Bmal1*^−/−^ mice. ^***^p < 0.001 by a two-tailed Student's *t*-test.

We next measured the circadian rhythm of spontaneous locomotor activity in *Nkx2.1-Bmal1*^−/−^ (*Nkx2.1-Cre;Bmal1*^*fl*/−^) mice. We examined two strains of mice, *Bmal1*^*fl*/−^ and *Nkx2.1-Cre;Bmal1*^+/*fl*^, as control mice. All three lines demonstrated a clear nocturnal pattern of behavioral rhythm in LD (Figure 3 and Figure S1). The free-running periods of behavioral rhythm in DD were similar between *Nkx2.1-Bmal1*^−/−^ and two strains of control mice (Figure 3C and Figure S1C). However, the activity was substantially reduced in *Nkx2.1-Bmal1*^−/−^ and *Nkx2.1-Cre;Bmal1*^+/*fl*^ mice compared to that of *Bmal1*^*fl*/−^ mice in LD (Figures S1A,E). We observed a similar attenuation of activity during the subjective night in those two strains free-running in DD (Figures S1A,D,E). Such results suggest that having the *Nkx2.1-Cre* allele caused a reduction of nocturnal activity regardless of the expression of *Bmal1*. Notably, the temporal pattern of nocturnal locomotor activity was clearly altered in *Nkx2.1-Bmal1*^−/−^ mice compared to both control strains (Figure S1A). Such an alteration was more obvious when daily activity profiles were adjusted in accordance with activity levels (Figure 3B and Figure S1B). In both LD and DD, control mice were more active during the first than the second half of the dark phase or subjective night. By contrast, *Nkx2.1-Bmal1*^−/−^ mice were more active during the second half of the dark phase or subjective night. The amplitude of the free-running rhythm of behavior, for which we calculated Qp values by periodogram analyses, was significantly lower in *Nkx2.1-Bmal1*^−/−^ mice than in control mice (Figure 3D and Figure S1D). Thus, *Nkx2.1-Bmal1*^−/−^ mice remained nocturnal, but did not maintain the precise temporal pattern of nocturnal activity. Because the function of SCN remained normal in those mice, peripheral circadian clocks in the extra-SCN hypothalamus might play a role in delineating the activity profile during the dark phase and subjective night.

### Altered temporal sleep-wakefulness pattern parallel to locomotor activity in *Nkx2.1-Bmal1*^−/−^ mice

**Figure 4:**
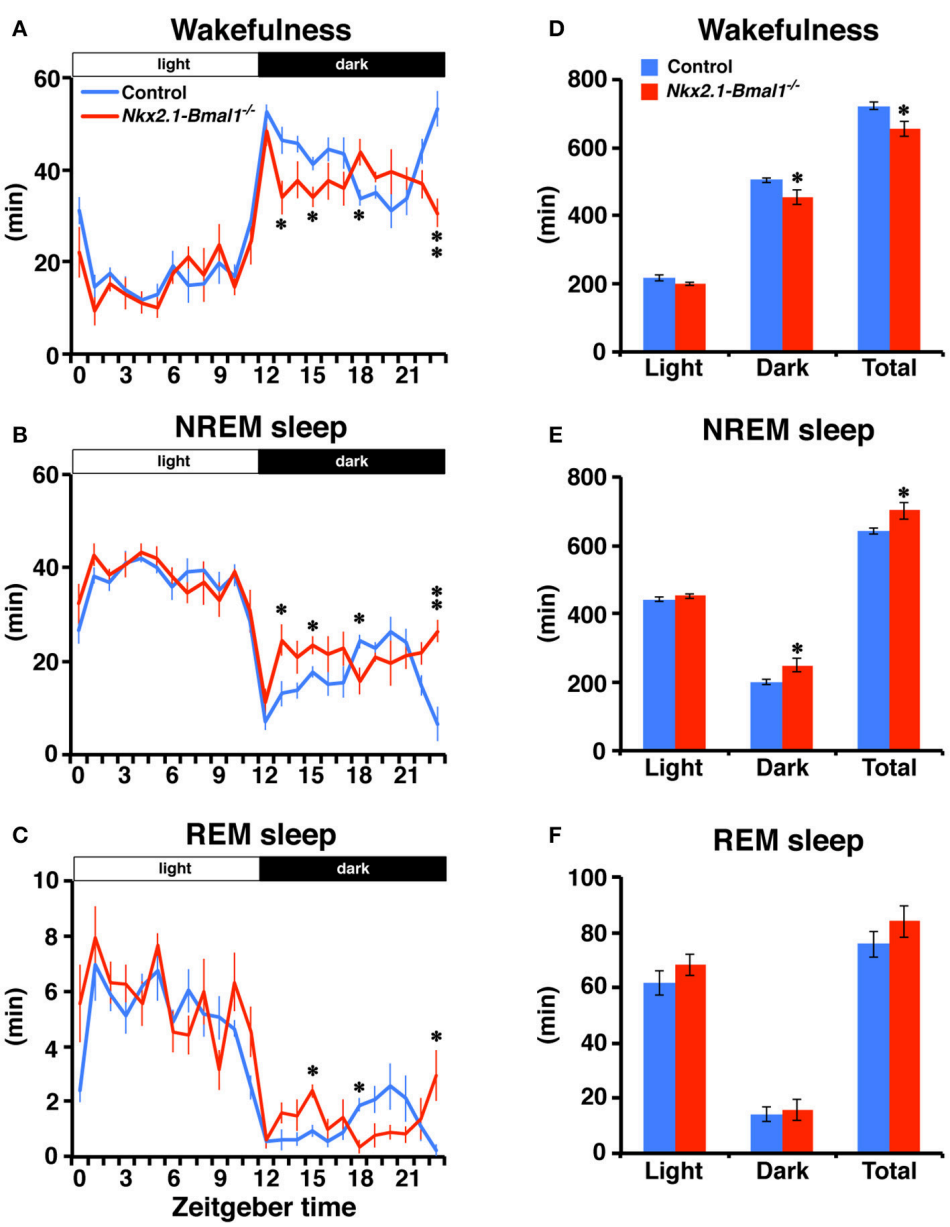
**Temporal pattern of sleep-wakefulness during the dark phase is altered in ***Nkx2.1-Bmal1***^**−/**^** ^−^ **mice. (A–C)** Hourly plots of time spent in wakefulness **(A)**, NREM sleep **(B)**, or REM sleep **(C)** in LD. **(D–F)** Time spent in wakefulness **(D)**, NREM sleep **(E)**, or REM sleep **(F)** during 12-h light phase (Light), 12-h dark phase (Dark), and 24-h day (Total). Values are mean ± SEM. *n* = 6 for control, *n* = 4 for *Nkx2.1-Bmal1*^−/−^ mice. ^*^p < 0.05; ^**^p < 0.01 by a Tukey-HSD *post-hoc*. The *p*-values calculated by two-way repeated measures ANOVAs are: **(A)** the effect of genotype, *F*_(1, 184)_ = 9.52, *p* = 0.015; the effect of time, *F*_(23, 184)_ = 31.71, *p* < 0.001; and the interaction between genotype and time, *F*_(23, 184)_ = 2.60, *p* < 0.001; **(B)** *F*_(1, 184)_ = 5.94, *p* = 0.041; *F*_(23, 184)_ = 29.30, *p* < 0.001; *F*_(23, 184)_ = 2.60, *p* < 0.001; **(C)** *F*_(23, 184)_ = 24.36, *p* < 0.001; *F*_(23, 184)_ = 2.26, *p* = 0.02; **(D)** the effect of genotype, *F*_(1, 8)_ = 9.52, *p* = 0.015; the effect of time (Light vs. Dark), *F*_(1, 8)_ = 559.33, *p* < 0.001; and the interaction between genotype and time, *F*_(1, 8)_ = 2.04, *p* = 0.191; **(E)** *F*_(1, 8)_ = 5.94, *p* = 0.041; *F*_(1, 8)_ = 567.63, *p* < 0.001; *F*_(1, 8)_ = 4.17, *p* = 0.075; **(F)** *F*_(1, 8)_ = 1.24, *p* = 0.299; *F*_(1, 8)_ = 155.87, *p* < 0.001; *F*_(1, 8)_ = 0.41, *p* = 0.538.

We also examined whether the temporal pattern of daily sleep-wakefulness was altered in *Nkx2.1-Bmal1*^−/−^ mice. As expected, *Nkx2.1-Bmal1*^−/−^ mice remained nocturnal, but the hourly patterns of wakefulness, NREM sleep, and REM sleep were all altered similarly to that of locomotor activity (Figure 4). Time spent in wakefulness during 24-h of day or during the 12-h dark phase were significantly reduced (whole day: 722.1 ± 10.7 vs. 654.6 ± 21.8 m, *p* = 0.015; dark phase: 505.1 ± 7.8 vs. 455.0 ± 21.6 m, *p* = 0.034), while those in NREM sleep were significantly increased in *Nkx2.1-Bmal1*^−/−^ mice (whole day: 642.3 ± 10.5 vs. 701.5 ± 25.9 m, *p* = 0.041; dark phase: 200.7 ± 7.5 vs. 249.4 ± 20.9 m, *p* = 0.034).

### Altered temporal pattern of nighttime food intake in *Nkx2.1-Bmal1*^−/−^ mice

**Figure 5:**
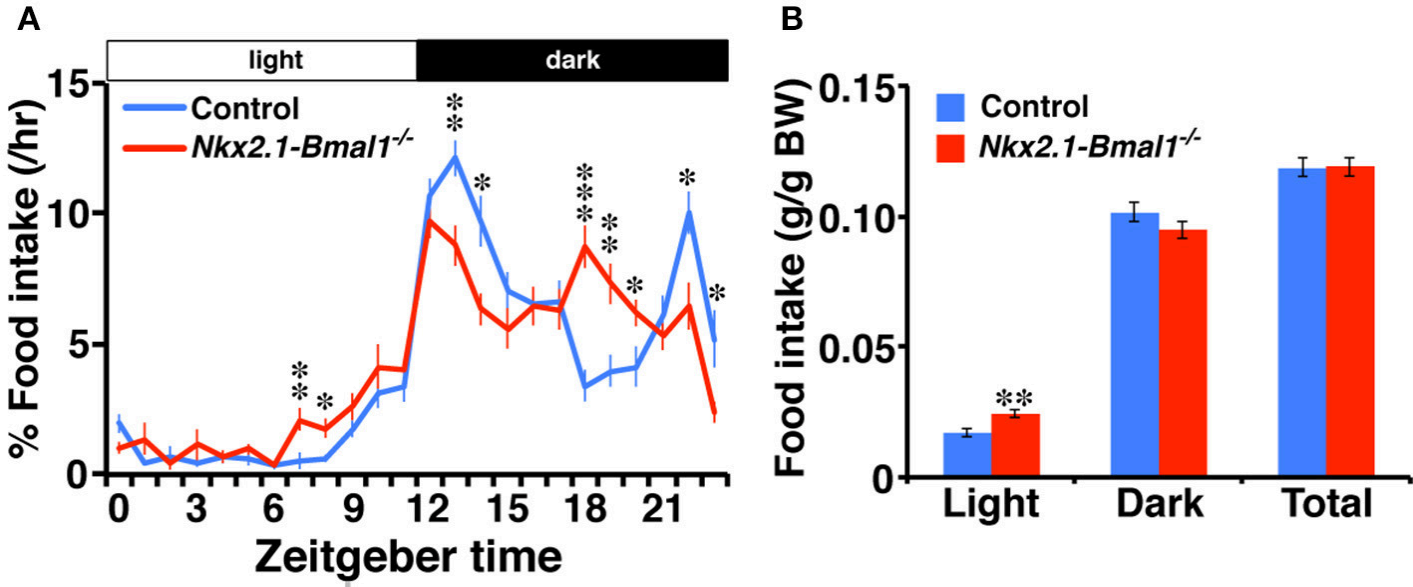
**Temporal pattern of food intake during the dark phase is altered in ***Nkx2.1-Bmal1***^**−/−**^ mice. (A)** Hourly plots of food intake, expressed as percentage of daily total. **(B)** Food intake per body weight during 12-h light phase (Light), 12-h dark phase (Dark), and 24-h day (Total). Values are mean ± SEM. *n* = 6 for control, *n* = 4 for *Nkx2.1-Bmal1*^−/^^−^ mice. ^*^p < 0.05; ^**^p < 0.01; ^***^p < 0.001 by a Tukey-HSD *post-hoc*. The *p*-values calculated by two-way repeated measures ANOVAs are: **(A)** the effect of genotype, *F*_(1, 529)_ > 1000, *p* < 0.001; the effect of time, *F*_(23, 529)_ = 53.22, *p* < 0.001; and the interaction between genotype and time, *F*_(23, 529)_ = 5.65, *p* < 0.001; **(B)** the effect of genotype, *F*_(1, 23)_ = 0.003, *p* = 0.960; the effect of time (Light vs. Dark), *F*_(1, 23)_ = 559.33, *p* < 0.001; and the interaction between genotype and time, *F*_(1, 23)_ = 2.04, *p* = 0.026.

We further examined the hourly food intake of *Nkx2.1-Bmal1*^−/−^ mice. As with the patterns of locomotor activity and sleep-wakefulness, the temporal pattern of food intake during the dark phase was altered in *Nkx2.1-Bmal1*^−/−^ mice (Figure 5). Daily food intake according to body weight did not differ between control and *Nkx2.1-Bmal1*^−/−^ mice (0.119 ± 0.003 vs. 0.119 ± 0.004 g/g body weight, *p* = 0.960). Intriguingly, food intake in the light phase was significantly increased in *Nkx2.1-Bmal1*^−/−^ mice compared to control mice (0.024 ± 0.002 vs. 0.017 ± 0.001 g/g body weight, *p* = 0.002), especially in the second half of the light phase. This increase might be correlated with a similar increase of locomotor activity in the latter half of the light phase (Figure 3A). As such, locomotor activity, sleep-wakefulness, and food intake demonstrated similar changes in their temporal patterns during the night in *Nkx2.1-Bmal1*^−/−^ mice.

## Discussion

We demonstrated that the SCN central clock alone cannot precisely delineate the locomotor activity pattern in the dark phase. The SCN grants mice nocturnality and roughly determines when they are active, whereas *Bmal1*-dependent local clocks in regions defined by developmental *Nkx2.1-Cre* expression might fine-tune the temporal pattern of nocturnal activity.

A remarkable feature of *Nkx2.1-Cre* mice in our study was that Cre expression occurred in most of the hypothalamus, but not in the SCN or its adjacent areas. Such a feature made this Cre driver line suitable for studying local circadian clocks in the hypothalamus without affecting the central clock in the SCN. During development, the SCN and PVH originate from a region immediately dorsal to an area marked by the expression of *Nkx2.1* and that generates many hypothalamic structures, including the VMH, DMH, ARC, and MN (Puelles and Rubenstein, 2003; Shimogori et al., 2010). In addition to the hypothalamus, *Nkx2.1* is developmentally expressed in the AEP and MGE, from which inhibitory neurons in the cortex and basal ganglia originate (Puelles and Rubenstein, 2003; Kessaris et al., 2006). Therefore, the distribution of βGal+ cells in adult *Nkx2.1-Cre;Rosa26R-lacZ* mice matched well with developmental *Nkx2.1* expression, except for those in the DRN, which might be due to an ectopic expression of Cre in *Nkx2.1-Cre* mice (Puelles and Rubenstein, 2003; Kessaris et al., 2006; Shimogori et al., 2010).

In contrast to those addressing peripheral clocks in peripheral organs, a limited number of reports address what local brain clocks do in the regulation of behavior and physiology. Dudley et al. initially reported that the lack of NPAS2, a paralog of CLOCK expressed in the forebrain, altered temporal patterns of locomotor activity and sleep-wakefulness in the active phase, meaning that a brief break in circadian locomotor activity beginning midway through the active phase was attenuated (Dudley et al., 2003). Pioneered by McClung et al. the contribution of local clocks in the ventral tegmental area and nucleus accumbens, which constitute the mesolimbic dopaminergic pathway, in mood regulation has been well-characterized (McClung et al., 2005; Roybal et al., 2007; Mukherjee et al., 2010; Spencer et al., 2013; Chung et al., 2014). More recently, Shimizu et al. reported that the suprachiasmatic nucleus circadian oscillatory protein (SCOP) mediates the circadian regulation of long-term memory formation by local clocks in the hippocampus (Shimizu et al., 2016), and Nakano et al., found that the same molecule regulates the circadian expression of anxiety-like behavior under the control of local clocks in the basolateral amygdala (Nakano et al., 2016). On another front, Yu et al. reported that *Bmal1*-dependent local clock in histaminergic neurons of tuberomammillary nucleus (TMN) regulate sleep architecture, likely via transcriptional control of the *histidine decarboxylase* gene, which encodes a histamine-synthesizing enzyme, but is not involved in regulating circadian rhythms (Yu et al., 2014). Furthermore, Orozco-Solis et al. revealed that the *Bmal1*-dependent local clock of VMH Sf1 neurons controls circadian energy expenditure through the rhythmic activation of BAT's metabolism, yet plays little role in delineating circadian activity and feeding rhythms (Orozco-Solis et al., 2016).

It is notable that circadian rhythms of locomotor activity, sleep-wakefulness, and food intake were all altered in parallel in *Nkx2.1-Cre* mice. Hypothalamic areas originating from *Nkx2.1*-positive neuroepithelium include several nuclei and neurons implicated in the regulation of locomotor activity, sleep-wakefulness, and feeding. For instance, orexinergic neurons in the perifornical area and histaminergic neurons in the TMN are wake-active and promote wakefulness, while a population of GABAergic neurons in the POA is sleep-active and increases sleep (Mieda and Sakurai, 2009; Saper et al., 2010). The basal forebrain also contains multiple types of neurons that control sleep-wakefulness (Saper et al., 2010; Xu et al., 2015). The Arc, VMH, DMH, and LHA play important roles in the regulation of feeding and energy metabolism (Gautron et al., 2015). As mentioned previously, mice without *Bmal1* specifically in histaminergic or VMH Sf1 neurons showed phenotypes unlike those of *Nkx2.1-Bmal1*^−/−^ mice (Yu et al., 2014; Orozco-Solis et al., 2016). Such observations suggest that *Bmal1*-dependent local clocks in histaminergic or Sf1 neurons are not solely responsible for altering nocturnal activity patterns in *Nkx2.1-Bmal1*^−/−^ mice. Orexinergic neurons may be well-placed to regulate multiple rhythms consistently because these neurons have been suggested to control locomotor activity, wakefulness, and food intake cooperatively (Mieda and Sakurai, 2009). Alternatively, multiple local brain clocks may differentially fine-tune these three rhythms.

Although, *Bmal1* is the sole non-redundant factor of cellular clocks, the impairment caused by loss of *Bmal1* may not be necessarily due to disruption of clock function. Indeed, several defects observed in standard *Bmal1* knockout mice, such as reduced life span, fertility, and body weight, were not detected in inducible *Bmal1* knockout mice that expressed the gene during embryogenesis but not after birth (Yang et al., 2016). Because *Nkx2.1* is expressed in the neuroepithelium during embryogenesis, the possibility that the impairments of circadian rhythms in *Nkx2.1-Bmal1*^−/−^ might reflect the function of *Bmal1* unrelated to the cellular clock cannot be excluded.

In conclusion, we demonstrated that *Bmal1*-dependent circadian clocks in the SCN and extra-SCN brain regions cooperate to delineate the precise daily patterns of locomotor activity, sleep-wakefulness, and food intake. However, the location of relevant local clocks remains unidentified and thus a task for future research.

## Author contributions

MM conceived and performed experiments, wrote the manuscript, and secured funding. EH performed experiments. MM and NK provided resources. TS provided expertise and feedback.

### Conflict of interest statement

The authors declare that the research was conducted in the absence of any commercial or financial relationships that could be construed as a potential conflict of interest.
