# Supplementary material for: Fine-Tuning Circadian Rhythms: The Importance of Bmal1 Expression in the Ventral Forebrain
Source: Front Neurosci. 2017 Feb 9;11:55. doi: 10.3389/fnins.2017.00055 (PMC5299009; doi:10.3389/fnins.2017.00055)
Supplement: Supplementary file 1 [file Image1.pdf]

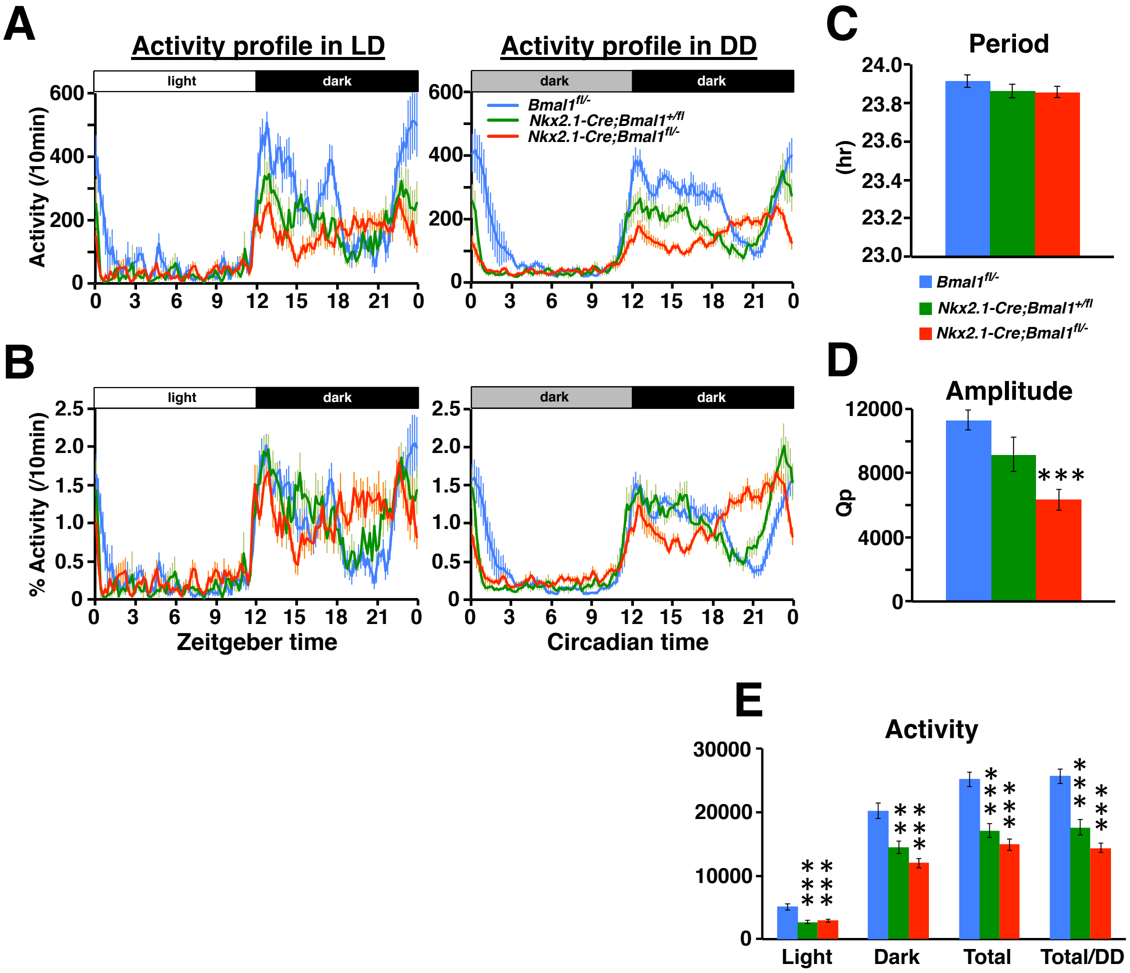

Figure S1. Comparison of locomotor activity between two control lines: *Bmal1*$^{fl/-}$ and *Nkx2.1-Cre;Bmal1*$^{+/fl}$ and *Nkx2.1-Cre;Bmal1*$^{fl/-}$ (*Nkx2.1-Bmal1*$^{-/-}$). (A, B) Daily profiles of locomotor activity in LD or DD shown with absolute counts (A) or expressed as percentage of daily total (B). (C, D) Mean free-running period and amplitude (Qp values of periodogram) in DD. Values for activity in DD were calculated for data on Days 5–19 in DD. (E) Activity during 12-h light phase (Light), 12-h dark phase (Dark), 24-h day in LD (Total), and a circadian period in DD (Total/DD). Values are mean ± SEM. n = 6 for *Bmal1*$^{fl/-}$, n = 8 for *Nkx2.1-Cre;Bmal1*$^{+/fl}$, n = 11 for *Nkx2.1-Cre;Bmal1*$^{fl/-}$ mice. $F(2,22) = 0.919$, $p = 0.414$ (C); $F(2,22) = 12.41$, $p < 0.001$ (D); $F(2,22) = 14.07$, $p < 0.001$ (E-Light); $F(2,22) = 19.51$, $p < 0.001$ (E-Dark); $F(2,22) = 27.09$, $p < 0.001$ (E-Total); $F(2,22) = 34.20$, $p < 0.001$ (E-Total/DD) by an one-way repeated measures ANOVA; *, $p < 0.05$; **, $p < 0.01$; ***, $p < 0.001$ as compared to *Bmal1*$^{-/fl}$ by a Tukey-HSD post-hoc test. The p values calculated by a two-way repeated measures ANOVA for the comparison of Light and Dark in (E) are: the effect of genotype, $F(2,22) = 24.38$, $p < 0.001$; the effect of time, $F(1,22) = 397.22$, $p < 0.001$; and the interaction between genotype and time, $F(2,22) = 8.50$, $p = 0.002$.
